# Biochemical comparison of two *Hypostomus* populations (Siluriformes, Loricariidae) from the Atlântico Stream of the upper Paraná River basin, Brazil

**DOI:** 10.1590/S1415-47572009000100008

**Published:** 2009-03-01

**Authors:** Kennya F. Ito, Erasmo Renesto, Cláudio H. Zawadzki

**Affiliations:** 1 Universidade Estadual de Maringá, Departamento de Biologia Celular e Genética, Maringá, PRBrazil; 2 Universidade Estadual de Maringá, Departamento de Biologia/Nupelia, Maringá, PRBrazil

**Keywords:** allozymes, *Hypostomus nigromaculatus*, fish genetics, genetic distance and polymorphism

## Abstract

Two syntopic morphotypes of the genus *Hypostomus* - *H. nigromaculatus* and *H.* cf. *nigromaculatus* (Atlântico Stream, Paraná State) - were compared through the allozyme electrophoresis technique. Twelve enzymatic systems (AAT, ADH, EST, GCDH, G3PDH, GPI, IDH, LDH, MDH, ME, PGM and SOD) were analyzed, attributing the score of 20 *loci*, with a total of 30 alleles. Six *loci* were diagnostic (*Aat-2*, *Gcdh-1*, *Gpi-A*, *Idh-1*, *Ldh-A* and *Mdh-A*), indicating the presence of interjacent reproductive isolation. The occurrence of few polymorphic *loci* acknowledge two morphotypes, with heterozygosity values *He* = 0.0291 for *H. nigromaculatus* and *He* = 0.0346 for *H.* cf. *nigromaculatus*. *F*_IS_ statistics demonstrated fixation of the alleles in the two morphotypes. Genetic identity (I) and distance (D) of Nei (1978) values were I = 0.6515 and D = 0.4285. The data indicate that these two morphotypes from the Atlântico Stream belong to different species.

## Introduction

The Neotropical region, encompassing southern Mexico and Central and South America, possesses the richest ichthyofauna in the world, with about 8,000 freshwater species (Schaefer, 1998). The order Siluriformes includes 34 families, 412 genera and more than 2,405 species (Nelson, 1994). Among the families belonging to this order, Loricariidae possesses more than 600 described species (Reis *et al.*, 2003), representing one of the largest families worldwide. This wide diversity has resulted in species identification problems, with many new species constantly being described (Pereira and Oyakawa, 2003; Cardoso and Silva, 2004). The family Loricariidae has been habitually divided into six subfamilies (Reis *et al.*, 2006). In the subfamily Hypostominae there are still many species that are not well defined, mainly due to wide intraspecific variation in morphology and color pattern. This happens mainly in the genus *Hypostomus* (Weber, 2003; Birindelli *et al.*, 2007; Jerep *et al.*, 2007). Biochemical markers are products of gene expression (proteins or secondary compounds) (*e.g.* isozymes). These are different molecular forms of an enzyme catalyzing the same reaction in the cell (Alfenas, 2006). Isozyme electrophoresis has been used with success to settle doubts regarding the taxonomic status of undescribed species of the Brazilian ichthyofauna (Renesto *et al.*, 2000, 2001, 2007; Zawadzki *et al.*, 2000, 2004).

Some specimens of *Hypostomus nigromaculatus* (Schubart, 1964) and a similar morphotype, called in this work *Hypostomus* cf. *nigromaculatus*, were collected in the Atlântico Stream, near Mandaguaçu, Paraná State, in the south of Brazil. *Hypostomus nigromaculatus* always presents distinct black spots on the body and fins, while *H*. cf. *nigromaculatus* usually presents clear gray spots, but possibly some dark spots, which made correct separation of the two morphotypes difficult, mainly in juveniles.

The main objective of the present work was to compare the electrophoretic patterns of syntopic samples of *Hypostomus nigromaculatus* and *Hypostomus* cf. *nigromaculatus,* in order to discover whether they belong to the same species, as well as to estimate the degree of genetic differentiation between them.

**Figure 1:**
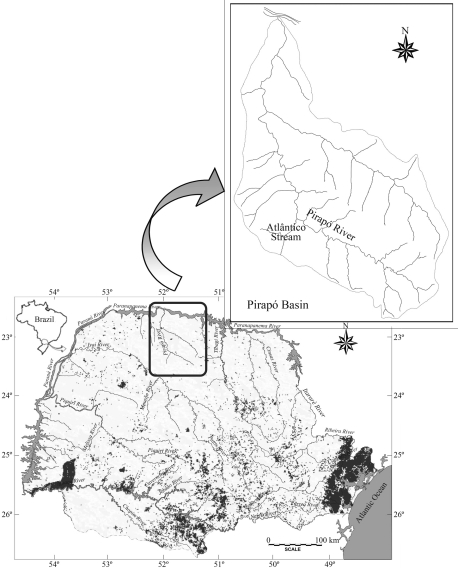
Paraná State hydrographic map. The small rectangle shows the location of the Pirapó River basin. The rectangle above shows the location of the Atlântico Stream in this basin.

**Figure 2:**
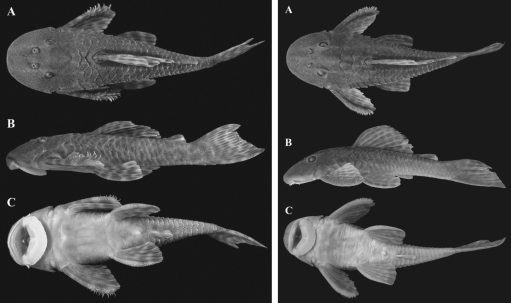
*Hypostomusnigromaculatus* (left, standard length = 75 mm) and *Hypostomus* cf. *nigromaculatus* (right, standard length = 101.3 mm)*.* A. Dorsal view. B. Lateral view. C. Ventral view.

**Figure 3:**
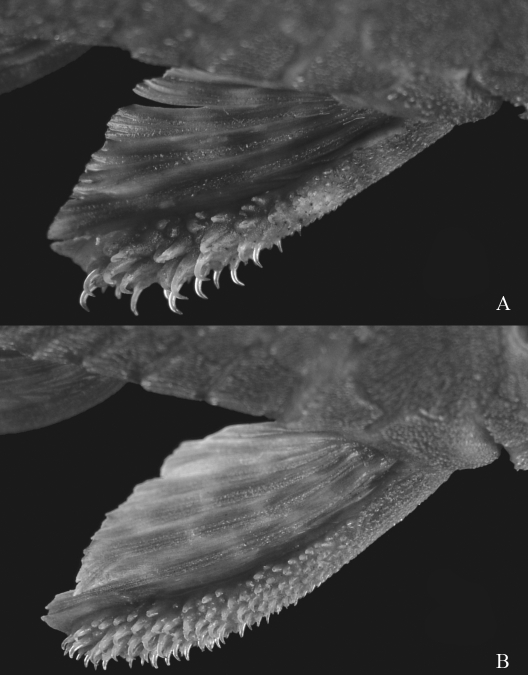
Pectoral fins. A. *Hypostomus nigromaculatus*. B. *Hypostomus* cf. *nigromaculatus*.

**Figure 4:**
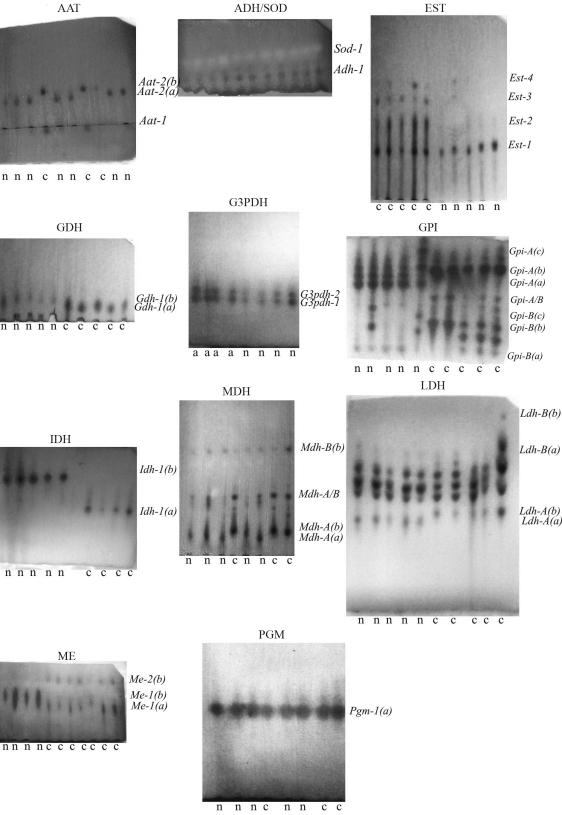
Electrophoretic patterns of 12 enzyme system analyses in corn starch gel for two morphotypes from the Atlântico Stream, Paraná State, Brazil. n = *Hypostomusnigromaculatus*; c = *Hypostomus* cf. *nigromaculatus*.

## Material and Methods

Specimens of *Hypostomus* were collected in the Atlântico Stream (Figure 1) (23°18'55" S; 52°00'55" W). The Atlântico Stream is a small stream branching from the Pirapó River, a tributary of the Paranapanema River (Mandaguaçu, northwestern Paraná State, southern Brazil). Thirty fish were collected, 15 of which were identified as *Hypostomus nigromaculatus* and 15 as *Hypostomus* cf. *nigromaculatus*, identification being based on morphological characters (Figure 2). All the specimens were collected on the same day and in the same place, by using a nylon thread cast net and conserved whole in liquid nitrogen. Samples of tissue from the muscles, liver, eyes, stomach, heart, kidneys and gills were homogenized with a plastic stick in propylene tubes (1.5 mL) with 100 μL of Tris-HCl 0.02 M, pH 7.5 buffer. Due to the presence of a great amount of fat in the liver, 100 μL of carbon tetrachloride (CCl_4_) was added to the tube (Pasteur *et al.*, 1988).

The enzyme extract was applied to the gel using Whatman 3 MM® paper wicks (4 mm x 8 mm) soaked with the samples, which were then submitted to continuous horizontal electrophoresis, under cooling. The gels were prepared with 15 g % of corn starch (Val *et al.*, 1981). Three buffer solutions were used: Tris 0.135 M/Citric acid 0.043 M pH 7.0 (TC), Tris 0.18 M/Boric acid 0.1/EDTA 0.004 M pH 8.6 (TBE) and Tris 0.1 M/Maleic acid 0.1 M/EDTA 0.01 M pH 7.4 (TEM). A voltage gradient of 60 V (measured in the extremities of the gel) was applied for 16 h. After electrophoresis, the gel was horizontally sliced length-wise into two slabs, which were incubated with specific staining solutions according to Murphy *et al.*, (1996).

Genetic variability was estimated by using Nei (1978) (He and Ho) average heterozygosity. The homogeneity of allele frequencies between populations was verified through a contingency chi-squared test. Unbiased genetic identity (I) and genetic distance (D) were also calculated according to Nei (1978). All of the estimates were calculated using POP GENE 1.31 software (Yeh and Boyle, 1997).

## Results

Tissues of *Hypostomus nigromaculatus* and *Hypostomus cf. nigromaculatus* were analyzed by corn starch gel electrophoresis using 12 enzymatic systems (Table 1). Twenty *loci* were detected (Table 2), presenting a total of 30 alleles. Figure 3 displays the electrophoretic pattern of each enzyme revealed for the two analyzed *Hypostomus* morphotypes. Six *loci* (*Aat-2, Gcdh-1, Gpi-A, Idh-1, Ldh-A and Mdh-A*) were diagnostic, *i.e.* they possess different alleles with 100% frequency in each morphotype.

The electrophoretic patterns of the 12 enzymatic systems were similar to those found by Zawadzki *et al.* (2001) for three species of *Hypostomus* from the Iguaçu River, except for esterase (EST), which had not been analyzed by these authors. The occurrence of few polymorphic *loci* was verified for both morphotypes: *Gpi-A and Gpi-B* for *H. nigromaculatus*, and *Ldh-B* and *Gpi-B* for *H.* cf. *nigromaculatus*.

Low polymorphism can also be verified by the effective number of alleles (Ae). As regards *H. nigromaculatus*, *Gpi-A* (Ae = 1.49) and *Gpi-B* (Ae = 1.30) were polymorphic, with an average of 1.04 ± 0.12 alleles per locus. As to *H.* cf. *nigromaculatus*, two *loci* were polymorphic, *Gpi-B* (Ae = 1.38) and *Ldh-B* (Ae = 1.64), with an average of 1.05 ± 0.16 alleles per locus.

We did not detect deviations from Hardy-Weinberg equilibrium at the polymorphic *loci* of *H. nigromaculatus* (p > 0.05).

The occurrence of few polymorphic *loci* was verified for the two morphotypes. Mean values of expected and observed heterozygosity for all the *loci* of *H.* cf. *nigromaculatus* were *He* = 0.0346 ± 0.1082 and *Ho* = 0.0167 ± 0.0745, while for *H. nigromaculatus* the values were *He* = 0.0291 ± 0.0911 and *Ho* = 0.0200 ± 0.0652.

Wright's (Wright, 1978) F statistics *F*_IS_ for estimating the excess of homozygotes was calculated for each locus. Mean values were *F*_IS_ = 0.312 for *H. nigromaculaus* and 0.455 for *H.* cf. *nigromaculatus*. The unbiased genetic identity (I) and genetic distance (D) of Nei (1978) were estimated as I = 0.6515 and D = 0.4285.

## Discussion

*H. nigromaculatus* had already been described by Schubart (1964), through specimens collected in the Mogí-Guaçu River, São Paulo State. Species of *Hypostomus* are widely distributed in medium or small streams of the upper Paraná River basin and morphological variations are commonly found among specimens of distant streams. Works on the genetics of these species are scarce in the literature. Rubert *et al.* (2008) found cytogenetic differences among populations of *H.nigromaculatus* from the Mogí-Guaçu River and streams of the Tibagi River basin. *H.nigromaculatus* and *H.* cf. *nigromaculatus* are both small-sized and have a dorso-ventrally depressed body, with relatively short pectoral and dorsal fins, and a large number of teeth and plates in the lateral areas of the abdomen. These similarities make correct identification difficult and indicate that they are probably phylogenetically similar species. However, *H. nigromaculatus* always presents evident dark spots on the body and fins, while *H*. cf. *nigromaculatus* usually presents light spots (not always evident) and sometimes some dark spots. In addition, *H. nigromaculatus* has a shorter standard length and smaller eyes than *H.* cf. *nigromaculatus*. There are also differences in relation to the pectoral fins, which are shorter and claviform in *H. nigromaculatus*, and the odontodes, which are more concentrated in the distal portion of the spine than in *H*. cf. *nigromaculatus* (Figure 4).

Studies of enzymatic *loci* have been used to verify the existence of species with doubtful taxonomic status or sibling species among sympatric morphotypes (Thorpe and Solé-Cava, 1994). Zawadzki *et al.* (2004), on studying *H. hermanni* and three morphotypes of *Hypostomus* collected in the Keller Stream, detected three diagnostic *loci* for *Hypostomus* sp. 1 (Gdh-A, G6pdh-A and G6pdh-B), eight for *Hypostomus* sp. 2 (sAta-B, G3pdh-A, G3pdh-B, Gpi-B, Ldh-A, Ldh-B, sMdh-B and sMdhp-A) and one for *Hypostomus* sp. 3 (sMdh-A), and so concluded that *Hypostomus* sp. 1, *Hypostomus* sp. 2 and *Hypostomus* sp. 3 were three different species.

In the present work, Glucose-6-phosphate isomerase (GPI) was the enzyme system which presented the largest observed heterozysosity (0.3425). With good expression in three tissues (gill, liver and muscle), this system presented two *loci:Gpi-A* with two alleles and *Gpi-B* with three. This enzyme can be used as a quick way of differentiating the two morphotypes, since *Gpi-A* is a diagnostic *locus*. Studies analyzing other fish of the family Loricariidae have also observed the existence of two *loci* for this same enzyme (Zawadzki *et al.*, 2000; Fisch-Muller *et al.*, 2001).

The occurrence of few polymorphic *loci* (10%) was verified for the two morphotypes. Inbreeding is a probable explanation for the low genetic variability in these *taxa*, as suggested by Zawadzki *et al.* (1999) for *Hypostomus derbyi* and *H. myersi* of the Iguaçu River (Paraná State, Brazil). Armored catfishes of the genus *Hypostomus* have sedentary habits, which lead to mating inside a family group, thus resulting in low genetic variability. On the other hand, inbreeding probably would not lead to fixation of alternative alleles in six *loci*. The fixed differences observed at the six loci are probably the result of drift fixation of different alleles over evolutionary time, before these different species became syntopic.

Factors that can cause the population not to be in Hardy-Weinberg equilibrium for a certain locus can be inbreeding, assortative mating and natural selection. In this case, we think the best hypothesis is inbreeding, since they are sedentary organisms, although we cannot caste aside possible gel-interpretation errors.

Nei's unbiased genetic identity (I) and genetic distance (D) indicate that one is dealing with two genetically different morphotypes. Taking into account the parameters proposed by Thorpe and Solé-Cava (1994), who analyzed the values of genetic identity obtained for different phylogenetic levels, populations that belong to the same species have I values superior to 0.85. On the other hand, for species belonging to the same genus, I values are between 0.35 and 0.85. Finally, for species belonging to a different genus, I values are inferior to 0.35. Nei's genetic identity between *H. nigromaculatus* and *H*. cf. *nigromaculatus* was I = 0.6515, thereby indicating they are different species of *Hypostomus.* Nei's genetic distance (Nei, 1978) corresponds to mean nucleotide substitutions per locus accumulated in the populations since they diverged from a common ancestor; *i.e.* substitution is proportional to evolutionary time (Dobzhansky *et al.*, 1977; Thorpe, 1982; Thorpe and Solé-Cava, 1994).

Even though they possess few morphological differences, there are significant genetic differences between the two morphotypes, thus showing reproductive isolation one from the other, and indicating that they belong to different *Hypostomus* species.

Paiva *et al.* (2005) demonstrated the existence of two undescribed species of *Hypostomus* in the Maringá Stream. They were caught just 10 km from the place where *H. nigromaculatus* and *H*. cf. *nigromaculatus* were collected, in the same river basin (Pirapó). Zawadzki *et al.* (2004) were also able to reveal the presence of three other undescribed species of *Hypostomus* in the Keller Stream, a tributary of the Ivaí River, 37 km from the Maringá Stream. Since six new *Hypostomus* species have been revealed to date in a small area of northwestern Paraná State, there should be many other undescribed species in the streams and rivers of the state. The ichthyofauna of Paraná State is poorly known. Therefore, we propose more widespread studies in the biochemical and molecular systematics of fish to improve our understanding.

## Figures and Tables

**Table 1:** Enzyme, Enzyme Commission number (EC n.), tissue, buffer and quaternary structure (QE) of enzymes analyzed in *Hypostomus*, *Hypostomusnigromaculatus* and *Hypostomus* cf. *nigromaculatus* from the Atlântico Stream (Paraná State, Brazil) by the corn-starch gel electrophoresis technique.

Enzymes	E C n.	Tissue	Buffer	QE
Alcohol dehydrogenase(ADH)	1.1.1.1	G/L	TBE/TEM	D
Aspartate aminotransferase (AAT)	2.6.1.1	L	TEM	D
Esterase (EST)	3.1.1.1	G/L	TBE/TEM	Mo
Glucose dehydrogenase (GCDH)	1.1.1.118	L	TEM	D
Glucose-6-phosphate isomerase (GPI)	5.3.1.9	G/L/M	TC	D
Glycerol-3-phosphate dehydrogenase (G_3_PDH)	1.1.1.8	M	TC	D
Isocitrate dehydrogenase -NADP+ (IDHP) EC 1.1.1.42	1.1.1.42	G/L/M	TC/TEM	D
L-Lactate dehydrogenase (LDH)	1.1.1.27	M	TC	T
Malate dehydrogenase -NAD^+^(MDH)	1.1.1.37	L	TC	D
Malate dehydrogenase -NADP^+^(ME)	1.1.1.40	L	TC	T
Phosphoglucomutase (PGM)	5.4.2.2	M	TC	Mo
Superoxide dismutase (SOD)	1.15.1.1	L	TBE/TEM	D

EC n. = Enzyme Commission number; G = gill; H = heart; L = liver; M = muscle; TEM = Tris/EDTA/Maleate pH 7.4 (Shaw and Prasad, 1970); TBE = Tris/EDTA/borate pH 8.6 (Boyer *et al.*, 1963); III TC = Tris/citrate pH 7.0 (Shaw and Prasad, 1970); D = dimeric; Mo = monomeric; T = tetrameric.

**Table 2:** Allele frequency and the homogeneity chi square test (χ^2^) for each of the 20 *loci* detected by the corn gel electrophoresis technique in *Hypostomus nigromaculatus* and *Hypostomus* cf. *nigromaculatus* from the Atlântico Stream (Paraná State, Brazil) (p = chi square probability).

*Locus*	Allele	*Hypostomus* *nigromaculatus*	*H.* cf. *nigromaculatus*	χ^2^	p
*Aat-1*	*a*	1.00	1.00	0.00	1.00
*Aat-2*	*a*	1.00	-	60.00	0.00
	*b*	-	1.00		
*Adh-1*	*a*	1.00	1.00	0.00	1.00
*Est-1*	*a*	1.00	1.00	0.00	1.00
*Est-2*	*a*	1.00	1.00	0.00	1.00
*Est-3*	*a*	1.00	1.00	0.00	1.00
*Est-4*	*a*	1.00	1.00	0.00	1.00
*Gdh-1*	*a*	-	1.00	36.00	0.00
	*b*	1.00	-		
*G3pdh-1*	*a*	1.00	1.00	0.00	1.00
*G3pdh-2*	*a*	1.00	1.00	0.00	1.00
*Gpi-A*	*a*	0.87	-	60.00	0.00
	*b*	-	1.00		
	*c*	0.13	-		
*Gpi-B*	*a*	0.80	0.17	39.60	0.00
	*b*	0.03	0.83		
	*c*	0.17			
*Idh-1*	*a*	-	1.00	60.00	0.00
	*b*	1.00	-		
*Ldh-A*	*a*	1.00	-	60.00	0.00
	*b*	-	1.00		
*Ldh-B*	*a*	1.00	0.73	9.23	0.00
	*b*	-	0.27		
*Mdh-A*	*a*	1.00	-	60.00	0.00
	*b*	-	1.00		
*Mdh-B*	*a*	1.00	1.00	0.00	1.00
*Me-1*	*a*	1.00	1.00	0.00	1.00
*Pgm-1*	*a*	1.00	1.00	0.00	1.00
*Sod-1*	*a*	1.00	1.00	0.00	1.00
